# Maternal immunity enhances *Mycoplasma hyopneumoniae* vaccination induced cell-mediated immune responses in piglets

**DOI:** 10.1186/1746-6148-10-124

**Published:** 2014-06-05

**Authors:** Meggan Bandrick, Kara Theis, Thomas W Molitor

**Affiliations:** 1 Veterinary Population Medicine, College of Veterinary Medicine, University of Minnesota, 1365 Gortner Ave, St. Paul, MN 55108, USA; 2 Present address: National Animal Disease Center, Agricultural Research Service, USDA, 1920 Dayton Ave, Ames, IA 50010, USA

**Keywords:** Maternal derived immunity, Cell-mediated immunity, Passive interference, *Mycoplasma hyopneumoniae*

## Abstract

**Background:**

Passively acquired maternal derived immunity (MDI) is a double-edged sword. Maternal derived antibody-mediated immunity (AMI) and cell-mediated immunity (CMI) are critical immediate defenses for the neonate; however, MDI may interfere with the induction of active immunity in the neonate, i.e. passive interference. The effect of antigen-specific MDI on vaccine-induced AMI and CMI responses to *Mycoplasma hyopneumoniae (M. hyopneumoniae*) was assessed in neonatal piglets. To determine whether CMI and AMI responses could be induced in piglets with MDI, piglets with high and low levels of maternal *M. hyopneumoniae-*specific immunity were vaccinated against *M. hyopneumoniae* at 7 d of age. Piglet *M. hyopneumoniae-*specific antibody, lymphoproliferation, and delayed type hypersensitivity (DTH) responses were measured 7 d and 14 d post vaccination.

**Results:**

Piglets with *M. hyopneumoniae-*specific MDI failed to show vaccine-induced AMI responses; there was no rise in *M. hyopneumoniae* antibody levels following vaccination of piglets in the presence of *M. hyopneumoniae*-specific MDI. However, piglets with *M. hyopneumoniae-*specific MDI had primary (antigen-specific lymphoproliferation) and secondary (DTH) *M. hyopneumoniae*-specific CMI responses following vaccination.

**Conclusions:**

In this study neonatal *M. hyopneumoniae*-specific CMI was not subject to passive interference by MDI. Further, it appears that both maternal derived and endogenous CMI contribute to *M. hyopneumoniae*-specific CMI responses in piglets vaccinated in the face of MDI.

## Background

Infectious disease is a major contributor to morbidity and mortality among infants, children, and other young animals. Ideally, strategies should be practiced at an early age to confer protection from infectious disease-related morbidity and mortality experienced later in life. Vaccination is a commonly used disease intervention strategy; however, there are many issues that complicate vaccinating neonates including vaccine safety and vaccine efficacy. Vaccination regimens may be unsuccessful in stimulating protective immunity in neonates due to both ontogenic immune immaturity and passively acquired maternal derived immunity (MDI) interfering with active immune development.

MDI is a critical contributor to the neonatal immune response. MDI is passively transferred to neonates across the placenta and via colostrum and milk in humans and mice [1-3] but only via colostrum and milk in pigs [4], horses [5], and cattle [6]. MDI is directly responsible for preventing or reducing the impact of infectious diseases in the neonate. In piglets MDI has been shown to be at least partially protective against many agents including *Escherichia coli*[7,8], *Mycoplasma hyopneumoniae* (*M. hyopneumoniae*) [9], Transmissible Gastroenteritis virus (TGE) [10], and Porcine Circovirus type 2 (PCV2) [11]. However, MDI may interfere with adaptive immune responses following vaccination, i.e. passive interference. For example, young pigs vaccinated in the face of antigen-specific MDI have suppressed antibody responses to *M. hyopneumoniae*[12], *Bordetella bronchiseptica*[13], Pseudorabies [14], Swine Influenza Virus (SIV) [15,16], and Classical Swine Fever (CSF) [17]. Further, while both cell-mediated immune (CMI) and antibody-mediated immune (AMI) mediators are transferred to neonates via colostrum, the amount of transferred maternal antibodies can often be correlated to the level of inhibition of neonatal AMI responses. Effects of maternal derived CMI on neonatal immune response development are less well understood. Whether neonatal CMI responses following vaccination or challenge are inhibited in the face of MDI is unclear.

Maternal CMI that is transferred to neonates across the placenta or in colostrum clearly participates in the neonatal CMI response. Transfer of functional tuberculin-specific immune cells, as evidenced by delayed-type hypersensitivity (DTH), has been demonstrated in infants from vaccinated mothers [18]. Transfer of CMI sensitivity to *Trichinella spiralis* and *Coccidioides immitis* antigens, as evidenced by antigen-specific proliferation and nematode load, and DTH, respectively, has been demonstrated in neonatal mice born to vaccinated dams [19,20]. Further, lymphocytes isolated from calves having received colostral cells from bovine viral diarrheal virus (BVDV) vaccinated mothers demonstrated greater CMI responsiveness to BVDV compared to lymphocytes isolated from calves receiving acellular colostrum [21]. We have shown that maternal colostral cells from *M. hyopneumoniae* vaccinated dams are transferred to piglets and participate in the *in vivo* neonatal CMI response to *M. hyopneumoniae* upon antigen challenge [22]. Still, it is unclear whether maternal derived CMI participates in immune interference or otherwise affects active CMI response development in the piglet. In other words, whether piglets vaccinated in the face of MDI develop antigen-specific CMI responses remains to be elucidated.

CMI is critical in the immune response to *M. hyopneumoniae*[23-26] and maternal *M. hyopneumoniae*-specific cells participate in the immune response following *M. hyopneumoniae* antigen challenge in piglets [22]. This study was conducted to test the hypothesis that piglets respond to *M. hyopneumoniae* vaccination with CMI but not AMI responses when vaccinated in the face of *M. hyopneumoniae*-specific MDI in a field setting. Piglets with *M. hyopneumoniae*-specific MDI failed to show vaccine induced AMI responses. In contrast, piglets with *M. hyopneumoniae*-specific MDI and vaccinated against *M. hyopneumoniae* developed primary and secondary *M. hyopneumoniae*-specific CMI responses.

## Results

### Sow response to vaccination

**Figure 1:**
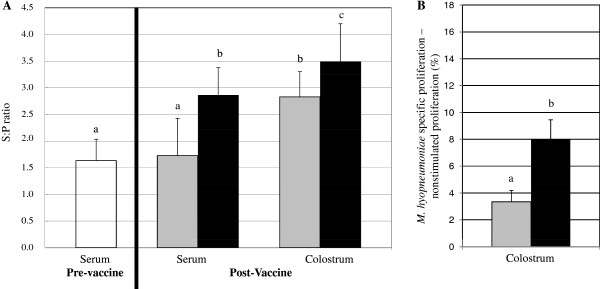
**Sow response *to Mycoplasma hyopneumoniae *vaccination.***M. hyopneumoniae* specific AMI (panel **A**) and CMI (panel **B**) were measured in sow blood and colostrum. Black bars represent vaccinated sows; gray bars represent nonvaccinated sows; white bars represent sows pre-vaccination; n = 10/group. Error bars are SEM; different subscripts represent significance at p < 0.05. *A. M. hyopneumoniae* specific antibody S:P ratios were measured in serum and colostrum of vaccinated and nonvaccinated sows using the Idexx HerdCheck^TM^ ELISA. *B. M. hyopneumoniae* specific CMI was measured in sow colostrum via lymphoproliferation. Antigen specific lymphoproliferation was determined by subtracting unstimulated (media only) lymphoproliferation from *M. hyopneumoniae* specific lymphoproliferation.

Prior to experimental vaccination, sows had *M. hyopneumoniae-*specific antibodies (Figure 1A) as expected due to prior on-farm vaccination. *M. hyopneumoniae* experimental vaccination of sows increased *M. hyopneumoniae*-specific AMI responses in blood and colostrum (Figure 1A) and *M. hyopneumoniae*-specific lymphoproliferative responses in colostrum (Figure 1B). Sows vaccinated in this study had significantly higher *M. hyopneumoniae*-specific serum antibody sample to positive (S:P) ratios compared to before vaccination (p = 0.0001) and compared to sows not vaccinated as part of this study (p = 0.002; Figure 1A). Similarly, experimentally vaccinated sows had significantly greater *M. hyopneumoniae*-specific antibody S:P ratios in colostrum compared to nonvaccinated sows (Figure 1A). Colostral mononuclear cells (CMC) from experimentally vaccinated sows demonstrated significantly greater *M. hyopneumoniae*-specific proliferation compared to CMC from nonvaccinated sows (p = 0.04; Figure 1B).

### Passive transfer of *M. hyopneumoniae*-specific immunity to piglets

**Figure 2:**
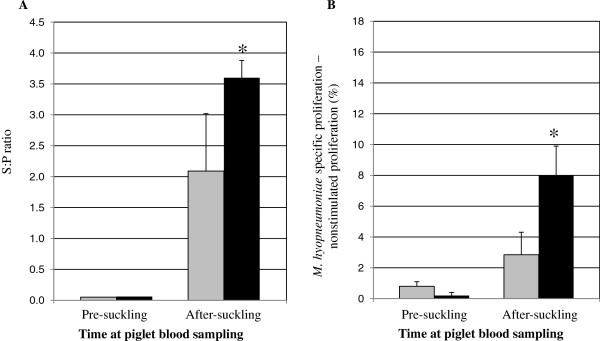
***M. hyopneumoniae *specific immunity is transferred to piglets.***M. hyopneumoniae* specific AMI (panel **A**) and CMI (panel **B**) were measured in piglet blood before (n = 7/group) and 24 hrs after (n = 10/group) colostrum ingestion. Black bars represent piglets from vaccinated sows; gray bars represent piglets from nonvaccinated sows. Error bars are SEM; *: p < 0.05. *A. M. hyopneumoniae*-specific antibody S:P ratios were measured in serum of piglets pre-suckling and 24 hrs after suckling using the Idexx HerdCheck^TM^ ELISA. *B. M. hyopneumoniae* specific CMI was measured in piglet blood via lymphoproliferation. Antigen-specific lymphoproliferation was determined by subtracting percent unstimulated lymphoproliferation (media only) from percent *M. hyopneumoniae*-specific lymphoproliferation.

Newborn piglets are naïve to *M. hyopneumoniae* (the swine placenta prohibits transfer of immune components *in utero*), and there is no evidence of transplacental infection with *M. hyopneumoniae*. Prior to colostrum ingestion piglets did not demonstrate *M. hyopneumoniae*-specific immunity, either AMI (Figure 2A) or CMI (Figure 2B). Following colostrum ingestion, piglets from vaccinated sows had significantly greater *M. hyopneumoniae* antibody S:P ratios compared to piglets from nonvaccinated sows (p = 0.0001; Figure 2A). Blood mononuclear cells (BMC) isolated from piglets of vaccinated sows 24 hr after suckling proliferated significantly more in response to stimulation with *M. hyopneumoniae* antigen compared to BMC isolated from piglets of nonvaccinated sows (p = 0.04; Figure 2B). There was no difference in *M. hyopneumoniae*-specific antibody S:P ratios or lymphoproliferation relative to piglet treatment group prior to piglet vaccination (data not shown).

### Piglet response to vaccination

**Figure 3:**
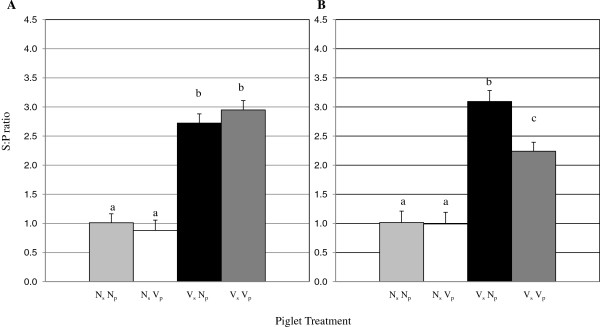
***M. hyopneumoniae *antibodies in piglet blood.***M. hyopneumoniae* specific antibody S:P ratios were measured in piglet blood 7 days post vaccination (dpv) (panel **A**) and 14 dpv (panel **B**) using the Idexx HerdCheck™ ELISA. Piglet treatment groups are as follows: N_s_ N_p_: nonvaccinated sow, nonvaccinated piglet; N_s_ V_p_: nonvaccinated sow, vaccinated piglet; V_s_ V_p_: vaccinated sow, vaccinated piglet; V_s_ N_p_: vaccinated sow, nonvaccinated piglet; n = 10/group. Error bars are SEM; different subscripts represent significance at p < 0.05.

*M. hyopneumoniae* vaccine was administered when piglets were 7 d of age. Piglets were vaccinated at 7 d of age in an attempt to stimulate endogenous immunity in the presence of transferred antigen specific MDI. *M. hyopneumoniae*-specific antibody levels did not differ among piglets from nonvaccinated sows relative to piglet vaccination status 7 days post vaccination (dpv; Figure 3A) or 14 dpv (Figure 3B). Two piglets that were considered *M. hyopneumoniae*-antibody negative based on S:P ratio did respond to vaccination with AMI responses by 14 dpv, though the magnitude of the response was not great enough to result in a difference between piglet groups. While the *M. hyopneumoniae*-specific antibody S:P ratio among piglets from nonvaccinated sows appeared lower at 7 dpv and 14 dpv compared to 24 hrs after suckling, this difference was not significant and there was no decrease 7 dpv to 14 dpv. *M. hyopneumoniae*-specific antibody S:P ratios among piglets from vaccinated sows were not different 7 dpv relative to piglet vaccination status (Figure 3A). There was no change in mean *M. hyopneumoniae*-specific antibody S:P ratio among nonvaccinated piglets from vaccinated sows (V_s_ N_p_) from 7 dpv to 14 dpv; however, vaccinated piglets from vaccinated sows (V_s_ V_p_) had lower *M. hyopneumoniae*-specific antibody S:P ratios at 14 dpv compared to 7 dpv (p = 0.05). V_s_ N_p_ piglets had greater *M. hyopneumoniae* antibody S:P ratios compared to V_s_ V_p_ piglets 14 dpv (Figure 3B).

**Figure 4:**
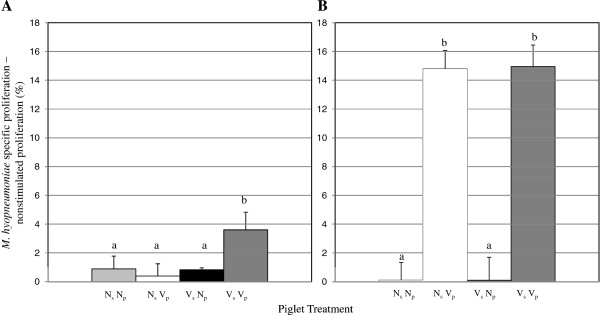
***M. hyopneumoniae*-specific proliferation. ***M. hyopneumoniae* specific proliferation by piglet blood mononuclear cells (BMC) was measured 7 days post vaccination (dpv) (panel **A**) and 14 dpv (panel **B**). Antigen-specific lymphoproliferation was determined by subtracting the percent unstimulated lymphoproliferation (media only) from the percent *M. hyopneumoniae* specific lymphoproliferation. Piglet treatment groups are as follows: N_s_ N_p_: nonvaccinated sow, nonvaccinated piglet; N_s_ V_p_: nonvaccinated sow, vaccinated piglet; V_s_ V_p_: vaccinated sow, vaccinated piglet; V_s_ N_p_: vaccinated sow, nonvaccinated piglet; n = 10/group. Error bars are SEM; different subscripts represent significance at p < 0.05.

To assess piglet *M. hyopneumoniae*-specific CMI *ex vivo*, *M. hyopneumoniae*-specific proliferation was assessed. Proliferative responses to concanavalin A (conA) were detected from BMC across piglets at both 7 dpv and 14 dpv (data not shown). Vaccination in the presence of MDI (V_s_ V_p_ piglets) resulted in earlier *M. hyopneumoniae*-specific proliferative responses (7 dpv) compared to all other groups of piglets (Figure 4A). Interestingly, BMC isolated from vaccinated piglets proliferated significantly more in response to stimulation with *M. hyopneumoniae* compared to BMC isolated from nonvaccinated piglets regardless of sow vaccination status 14 dpv (p < 0.01; Figure 4B). There was no difference in proliferation by BMC isolated from N_s_ V_p_ compared to V_s_ V_p_ piglets at 14 dpv. BMC isolated 14 dpv from V_s_ V_p_ piglets proliferated more in response to stimulation with *M. hyopneumoniae* than BMC isolated from the same group of piglets at 7 dpv. Similarly, *M. hyopneumoniae-*specific proliferative responses from N_s_ V_p_ piglets at 14 dpv were significantly greater than those from the same group of piglets at 7 dpv.

**Figure 5:**
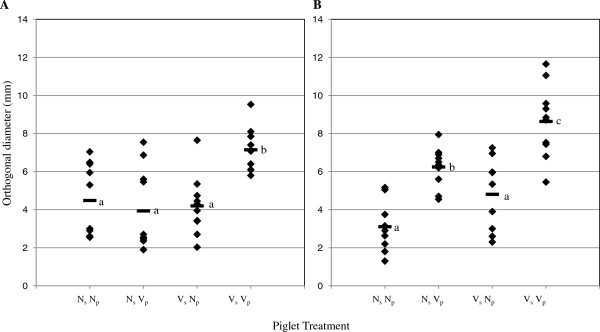
***M. hyopneumoniae*-specific DTH responses. ***M. hyopneumoniae* specific DTH responses were measured 7 days post vaccination (dpv) (panel **A**) and 14 dpv (panel **B**) in piglets. Each diamond represents an individual animal; horizontal bars represent sample means. Piglets were DTH tested at one time point only; n = 10/group. Piglet treatment groups are as follows: N_s_ N_p_: nonvaccinated sow, nonvaccinated piglet; N_s_ V_p_: nonvaccinated sow, vaccinated piglet; V_s_ V_p_: vaccinated sow, vaccinated piglet; V_s_ N_p_: vaccinated sow, nonvaccinated piglet. Different subscripts represent significance at p < 0.05.

To assess piglet *M. hyopneumoniae*-specific CMI *in vivo*, DTH testing was performed. In control DTH tests, piglets across treatment groups responded to phytohemagglutinin (PHA) and none responded to saline at both injection times (7 dpv and 14 dpv; data not shown). *M. hyopneumoniae*-specific DTH lesions were detected in some piglets across all treatment groups at both time points (Figure 5). V_s_ V_p_ piglets had significantly larger *M. hyopneumoniae*-specific DTH lesions (mean orthogonal diameter) compared to all other treatment groups at 7 dpv (p < 0.01; Figure 5A). *M. hyopneumoniae*-specific DTH lesions among nonvaccinated piglets did not differ in orthogonal diameter 14 dpv (Figure 5B). The mean orthogonal diameter of *M. hyopneumoniae*-specific lesions of nonvaccinated piglets was smaller than that of N_s_ V_p_ piglets (p = 0.05) and V_s_ V_p_ piglets (p = 0.01) (Figure 5B) 14 dpv. In contrast to proliferative responses, *M. hyopneumoniae*-specific DTH lesions of V_s_ V_p_ piglets were significantly larger than that of N_s_ V_p_ piglets (p = 0.01) 14 dpv. The mean orthogonal diameter of *M. hyopneumoniae*-specific DTH lesions was significantly larger at 14 dpv compared to 7 dpv for N_s_ V_p_ piglets only.

## Discussion

Maternal derived antibodies and lymphocytes acquired across the placenta or postnatally in colostrum or milk are critical participants in the neonatal immune response. Neonatal vaccination has been practiced across a variety of antigens in an effort to confer protection from specific pathogens during the period in which the neonatal immune system matures and maternal antibodies wane. However, MDI may interfere with immune priming and the generation of memory responses following neonatal vaccination.

This study was conducted to determine whether neonatal piglets respond with AMI and/or CMI responses to *M. hyopneumoniae* vaccination when vaccinated in the face of maternal *M. hyopneumoniae*-specific immunity. To determine whether CMI and AMI responses could be induced in piglets with MDI, piglets from *M. hyopneumoniae* vaccinated and nonvaccinated dams were vaccinated against *M. hyopneumoniae* at 7 d of age. Piglet *M. hyopneumoniae-*specific antibody, lymphoproliferative, and DTH responses were measured 7 dpv and 14 dpv. Vaccination of piglets from vaccinated sows did not induce AMI responses in those piglets; there was no rise in *M. hyopneumoniae* S:P ratios following piglet vaccination. In contrast, evidence from both antigen-specific proliferation and DTH testing demonstrates that *M. hyopneumoniae*-specific CMI priming and anamnestic responses are induced following vaccination of piglets with *M. hyopneumoniae*-specific MDI. Taken together, neonatal piglet *M. hyopneumoniae*-specific CMI responses induced by vaccination are not (at least wholly) inhibited by passive interference with *M. hyopneumoniae*-specific MDI.

In the present study, there is no evidence that piglets vaccinated in the face of MDI developed active *M. hyopneumoniae*-specific AMI responses within 14 dpv. These results are in agreement with Hodgins et al. [12] who showed no rise in serum *M. hyopneumoniae* antibody titer 9 weeks post vaccination in piglets vaccinated at 14 d of age in the presence of high levels of antigen-specific MDI [12]. The lack of rise in *M. hyopneumoniae* antibody S:P ratios in piglets vaccinated in the face of MDI is most likely due to passive interference; however, this cannot be concluded with certainty in the current study as a group of vaccinated piglets lacking *M. hyopneumoniae-*specific maternal immunity was not included. However, piglets with low levels of maternal *M. hyopneumoniae-*specific AMI at the time of vaccination did produce *M. hyopneumoniae-*specific antibodies following vaccination, indicating that vaccination can induce AMI responses in 7 d old pigs dependent upon the level of maternal antigen-specific AMI in agreement with [12]. It is possible that more piglets responded to *M. hyopneumoniae* vaccination with IgM responses; however, the ELISA test used lacked isotype specificity and IgG likely overshadowed any IgM response in all samples tested. While including a group of SPF piglets may have resulted in more clear interference results, SPF piglets are not representative of most commercial pigs. Further, as *M. hyopneumoniae* vaccination is one of the most commonly used vaccines across swine farms [27], the data in this report represent field conditions. It is unclear why *M. hyopneumoniae* S:P ratios were greater in V_s_ N_p_ piglets compared to V_s_ V_p_ piglets at 14 dpv; this difference may be attributable to inter-animal variation.

There is a plethora of data demonstrating passive interference by maternal immunity with neonatal AMI responses following vaccination; however, reports assessing CMI responses following neonatal vaccination in the face of MDI are limited. Not only is there a paucity of information regarding the neonatal CMI response to vaccination in the face of MDI, the information that does exist is largely restricted to *in vitro* studies. For example, antigen specific proliferative responses and cytokine production have been demonstrated in infants vaccinated against measles virus [28], and neonatal mice vaccinated against measles virus [29,30] and lymphocytic choriomeningitis virus [31] following vaccination in the face of antigen-specific MDI. Notably, humans and mice are exposed to MDI during gestation, and the detection of antigen-specific reactivity in the neonate may be a result of antigen priming while *in utero*[32,33]. The lack of exposure to MDI prenatally make pigs, cattle, and horses excellent models to study the role of colostral immunity in the neonate, yet few reports have investigated CMI following neonatal vaccination of these species. The current study shows that piglets with MDI respond to *M. hyopneumoniae* vaccination with antigen specific CMI responses, and unlike previous studies, we show evidence of CMI stimulation *in vivo*. DTH testing is a valuable measure of CMI since the DTH test is an *in vivo* test and DTH responses are by definition anamnestic responses.

In agreement with the present study, Bouma et al. [34] showed that 3-week-old piglets challenged with pseudorabies virus developed virus-specific proliferative responses regardless of maternal pseudorabies status. Further, piglets with pseudorabies-specific MDI and vaccinated against pseudorabies had lower pseudorabies virus antibody titers than their counterparts without pseudorabies-specific MDI [34]. Similarly, calves vaccinated against BVDV in the face of MDI developed CMI responses and did not show evidence of anamnestic AMI responses upon re-exposure to the antigen [35]. Interestingly, calves vaccinated against BVDV are protected from disease even in the absence of a specific AMI response [35,36]. Therefore, just because neonates fail to develop detectable anamnestic AMI responses following vaccination in the face of MDI does not signify that there is an inhibition of the immune response. Rather, vaccination may induce CMI responses that are protective. Collectively these studies show that neonates are capable of generating CMI responses in the face of MDI and that passively transferred MDI may not interfere with neonatal CMI.

Neonatal vaccination in the face of passive immunity does not always result in CMI responses. The capacity for neonates with MDI to generate CMI responses following vaccination may depend on their age and immune maturation at vaccination. Further, the concentration of maternal AMI at the time of vaccination may play a role in the neonatal immune response since the level of interference may be dependent on the level of maternal AMI in the neonate [37]. For example, piglets vaccinated against SIV [16] or CSF [38] do not experience CMI stimulation when vaccinated in the face of high levels of antigen-specific maternal AMI but do show evidence of CMI stimulation following vaccination when levels of maternal antibodies are low. Further, the capacity for neonates to generate specific CMI responses in the face of MDI may be reliant on the administration method of the vaccine. For example, piglets with MDI specific for and vaccinated against pseudorabies shed less virus when vaccine was administered intranasally compared to intramuscularly [39]. Vaccine type or immunogenicity may also influence the neonate’s response to vaccination. DNA vaccines are an especially alluring neonatal vaccination paradigm since they replicate *in vivo* but do not pose a risk of infection. Some DNA vaccines induce immune responses in neonates when administered in the face of MDI [29,40,41] while others do not [29,42,43]. Further, adjuvants that enhance CMI or antigen presentation (such as CpG motifs) may help the neonatal immune system overcome the opposing effects of maternal AMI [44].

Differences in neonatal responses to vaccination may also be due to the presence of functional maternal lymphoid cells at the time of vaccination. Various studies have shown that transferred maternal cells augment the neonatal response to nonspecific [45,46] and specific [18-20,22] antigens. In the current study piglets vaccinated against *M. hyopneumoniae* in the face of *M. hyopneumoniae*-specific MDI (V_s_ V_p_ piglets) exhibited greater *M. hyopneumoniae* specific CMI responses 7 dpv compared to all other groups. Therefore, CMI priming and anamnestic responses at 7 dpv depended upon piglet vaccination and transferred antigen-specific MDI. Capozzo et al. [29] showed that neonatal mice vaccinated against measles virus in the face of measles virus-specific MDI exhibited greater measles-specific CMI responsiveness (IFN-γ production and lymphoproliferation) compared to neonatal mice vaccinated against measles but without virus-specific MDI. Taken together, these studies suggest that MDI plays a role in the vaccine-induced CMI response detected in neonates.

Capozzo et al. [29] suggested that the CMI responses observed in mice vaccinated against measles virus in the face of measles virus-specific MDI was due to neonatal T cells that were stimulated by endogenous dendritic cells that had engulfed maternal antibodies bound to measles antigens [29]. In support of the idea proposed by Capozzo et al. that neonatal cells are responding to vaccine antigen, in this study, since DTH lesions and proliferative responses were greater in N_s_ V_p_ piglets compared to N_s_ N_p_ piglets 14 dpv, it is reasonable to conclude that piglet derived T cells are contributing to the neonatal response to *M. hyopneumoniae* antigen. In addition to neonatal cells responding to vaccination, we suggest that the CMI responses observed at 7 dpv are also due to the functional activity (proliferation or secretion of soluble immune mediators, e.g. cytokines) of transferred antigen-specific maternal lymphocytes that are restimulated via neonatal vaccination. We have previously shown that maternal lymphocytes primed during vaccination are transferred to and are functional in piglets [22]. Here we show that transferred maternal cells may be stimulated via piglet vaccination—at 7 dpv lymphoproliferative and DTH responses by V_s_ V_p_ piglets were greater than those of N_s_ V_p_ piglets. Further research is required to determine if these responses are protective.

Evidence suggests that maternal microchimerism (retained maternal cells or DNA) persists into adulthood [47]; however, it is unclear for how long maternal cells are functional in the offspring. In terms of strategizing vaccination regimens to take advantage of both endogenous and passive immunity, the persistence of functional activity of maternal CMI in the recipient warrants elucidation. Whether the persistence of function rather than merely presence (anergy) of maternal CMI in the recipient requires antigen stimulation is to be determined.

## Conclusion

In the present study, we showed that maternal *M. hyopneumoniae*-specific AMI and CMI is transferred to and detectable in piglets. Further, transferred maternal *M. hyopneumoniae*-specific CMI is functional and works in concert with vaccine stimulated endogenous *M. hyopneumoniae*-specific CMI to respond to *M. hyopneumoniae* in the piglet. We also demonstrated that vaccination was sufficient to prime a 7 day-old piglet’s immune system. Piglets developed *M. hyopneumoniae*-specific CMI responses when vaccinated in the face of MDI. In conclusion, vaccination of neonatal pigs against *M. hyopneumoniae* in the face of antigen-specific MDI results in CMI priming and anamnestic CMI responses following subsequent exposure to *M. hyopneumoniae* antigen.

## Methods

### Animals

This study was approved by the University of Minnesota’s Institutional Animal Care and Use Committee and all animals were cared for and housed under the University of Minnesota’s Institutional Animal Care and Use Committee guidelines. At all times during the study, animals were housed at a commercial facility (Prairie Land Pork, Nicollet, MN) known to be porcine reproductive and respiratory syndrome virus negative. According to the facility’s protocol, replacement gilts were vaccinated against *M. hyopneumoniae* (Myco Silencer, Intervet, Whitehouse Station, NJ, USA) at days 17 and 45 of gilt acclimatization by farm staff; the total acclimatization period was 110 days. Randomly selected piglets from the farm were tested for *M. hyopneumoniae* by PCR following nasal swabbing prior to starting the present study [48]. *M. hyopneumoniae*-specific PCR testing was performed using a previously described protocol [49]. All swabs were negative for *M. hyopneumoniae* DNA, indicating that *M. hyopneumoniae* was not circulating on the farm.

Animals were chosen to participate in this study based on breeding date and on gilt and first parity sow status. An equal number of gilts and first parity sows (from here known as sows) were randomly stratified into one of two groups: boosted or primed, based on previous on-farm vaccination. Boosted animals were vaccinated as part of this study and are referred to as “vaccinated” while primed animals were not vaccinated as part of this study and are referred to as “nonvaccinated.” Experimental vaccination was against *M. hyopneumoniae* (Respisure-One®, Pfizer Animal Health, Kalamazoo, MI, USA), an adjuvanted bacterin. Respisure-One®, labeled for single dose administration in healthy swine one day of age or older, was given (2 ml intramuscular) at 5 and 3 weeks prior to the anticipated farrowing date.

Farrowings were monitored and piglets were ear-tagged at birth. To ensure piglets received both maternal CMI and AMI, no cross-fostering was practiced among study animals [50]. Eighty piglets (n = 3 per sow) were chosen based on sow vaccination status. Twenty piglets from vaccinated sows and 20 piglets from nonvaccinated sows were selected randomly and immunized with the same vaccine as given to the sows. Piglet vaccine was administered at 7 d according to manufacturer guidelines and resulted in four treatment groups of 20 piglets each as follows: (1) sow vaccination, piglet vaccination (V_s_ V_p_); (2) sow vaccination, piglet nonvaccination (V_s_ N_p_); (3) sow nonvaccination, piglet vaccination (N_s_ V_p_); and (4) sow nonvaccination, piglet nonvaccination (N_s_ N_p_).

### Sampling

Blood collection of sows occurred at 5 weeks prepartum and 3 weeks postpartum. Blood was collected from the jugular vein into EDTA Vacutainer® tubes (BD, Franklin Lakes, NJ, USA). Colostrum was collected from all sows within 2 hrs of farrowing. Teats were scrubbed with alcohol wipes (70%; Medline, Mundelein, IL, USA) and gloves were worn to minimize sample contamination. Colostrum (25 ml) was collected manually from all functional teats.

Blood was collected from piglets before colostrum ingestion, 24 hr after colostrum ingestion, and at 14, and 21 days of age. Blood was collected via jugular venipuncture into sterile EDTA Vacutainer® tubes. Blood sampling times are expressed in terms of days post vaccination (dpv).

### Laboratory methods

#### Mononuclear cell isolation and stimulation

Mononuclear cells were isolated from sow and piglet blood via Ficoll density centrifugation as described [51] with modifications. Piglet blood was diluted 1:2 in sterile PBS prior to layering on lymphocyte separation media to improve cell recovery yield. Mononuclear cells were isolated from colostrum as described [52]. Cells were microscopically enumerated and viability was assessed via Trypan Blue exclusion. Viability of blood mononuclear cells (BMC) was at least 95% and viability of colostral mononuclear cells (CMC) was at least 90%.

A dye-dilution method was used in order to evaluate the lymphoproliferative response to *M. hyopneumoniae*. Cells were stained with the membrane stain carboxyfluorescein diacetate succinimidyl ester (CFSE; 5 μM; ICT, Bloomington, MN, USA) and washed with RPMI supplemented with 10% FBS, 100 U penicillin G per ml, and 100 μg of streptomycin per ml to stop the reaction. Cells were resuspended in RPMI and plated in duplicate at 5×10^5^ cells/well in 200 μl in round bottom 96-well plates. CMC and BMC were stimulated with 10 μg/ml *M. hyopneumoniae* antigen as described [26]. The *M. hyopneumoniae* antigen was prepared as described [22]; *M. hyopneumoniae* at passage 15 was harvested by continuous flow centrifugation at 70,000 x g and resuspended in Tris-sodium chloride buffer. *M. hyopneumoniae* was inactivated by one freeze-thaw cycle and then by sonic disruption. Nonstimulated and ConA (5 μg/ml; Sigma-Aldrich, St. Louis, MO, USA) stimulated cultures served as negative and positive controls, respectively. Experimental, negative, and positive controls were analyzed for each animal.

Following 5 d incubation, cells were transferred to sterile Facs tubes and washed. Proliferation was analyzed by flow cytometry using a Facs Caliber flow cytometer (Becton Dickinson Immunocytometry System, San Jose, CA, USA). Non-stained, non-stimulated cells and stained, non-stimulated cells were used to establish a baseline for the proliferation assay. Event acquisition was set for 10,000 events in a region encompassing the CFSE-positive quadrant. Results were analyzed by BD Cellquest™ Pro software (BD Biosciences, San Jose, CA, USA). *M. hyopneumoniae*-specific proliferation data are described as the percent *M. hyopneumoniae-*stimulated proliferation - nonstimulated proliferation.

### DTH testing

DTH testing was used as an *in vivo* measure of the CMI response. The DTH assay for *M. hyopneumoniae* was performed as originally applied [23] with modifications. The *M. hyopneumoniae* antigen used in the DTH assay was the same antigen as used in the *in vitro* proliferation assay. *M. hyopneumoniae* antigen (300 μg/ml in 0.1 ml physiological saline) was injected intradermally in 10 piglets per group at 14 d of age and a second set of 10 piglets per group at 21 d of age. PHA (20 μg/ml in 0.1 ml physiological saline; Sigma, St. Louis, MO, USA) and physiological saline (0.1 ml) were used as positive and negative controls, respectively. Injections were performed in the inguinal region. Injection sites were clearly marked with livestock paint. The DTH injection sites were assessed immediately and 36 h post injection. DTH lesion diameters were measured with digital calipers; DTH data are shown as mean orthogonal diameter of induration. Piglets were subject to DTH testing only once to avoid the possibility of the DTH antigens influencing subsequent DTH test results.

### Antibody measurement

*M. hyopneumoniae*-specific antibodies were measured in all blood and colostrum samples employing Idexx ELISA kits (IDEXX Laboratories, Westbrook, Maine, USA) as described [53]. The Idexx *M. hyopneumoniae* ELISA kit is isotype non-specific. Positive and negative *M. hyopneumoniae* serological status was determined based on optical density (OD) of the sample to positive ratio (S:P); S:P = (sample OD - negative control OD)/(positive control OD - negative control OD). All samples were run in duplicate and sample means were used to determine the final S:P ratio. S:P ratios ≥0.4 were classified as positive; S:P ratios <0.4 were classified as negative.

### Data analysis and statistics

Normality of data was evaluated prior to statistical analysis and approximated to be normal. The difference in *M. hyopneumoniae*-specific antibody S:P ratio in sow sera before and after vaccination, the difference between *M. hyopneumoniae*-specific antibody S:P ratios in sera and colostrum of vaccinated or nonvaccinated sows, and the difference between *M. hyopneumoniae*-specific proliferation by CMC isolated from vaccinated or nonvaccinated sows was analyzed by the student *t*-test. The effect of piglet vaccination on *M. hyopneumoniae*-specific AMI and CMI responses (piglet *M. hyopneumoniae*-specific antibody S:P ratios, *M. hyopneumoniae*-specific proliferation, and *M. hyopneumoniae*-specific DTH lesion size) was analyzed via two-way ANOVA with piglet vaccination and sow vaccination being the two factors. Comparisons found to be significant by ANOVA (p < 0.05) were analyzed by Tukey’s HSD method. Student *t*-test was used to analyze the difference in mean *M. hyopneumoniae*-specific DTH lesion orthogonal diameter and the difference in *M. hyopneumoniae*-specific antibody S:P ratios between 7 dpv and 14 dpv among piglets of the same group. Statistical analysis was performed using GraphPad Prism 5 (Graph Pad Software, Inc, CA, USA).

## Abbreviations

AMI: Antibody-mediated immunity; BMC: Blood mononuclear cells; BVDV: Bovine viral diarrheal virus; CMI: Cell mediated immunity; conA: Concanavalin A; CSF: Classical swine fever; dpv: Days post vaccination; DTH: Delayed type hypersensitivity; *M. hyopneumoniae*: *Mycoplasma hyopneumoniae*; MDI: Maternal derived immunity; PCV2: Porcine circovirus type 2; PHA: Phytohemagglutinin; S:P: Sample to positive; SIV: Swine influenza virus; TGE: Transmissible gastroenteritis virus; CMC: Colostral mononuclear cells; N_s_ V_p_: Nonvaccinated sow, vaccinated piglet; V_s_ V_p_: Vaccinated sow, vaccinated piglet; V_s_ N_p_: Vaccinated sow, nonvaccinated piglet; N_s_ N_p_: Nonvaccinated sow, nonvaccinated piglet.

## Competing interests

None of the authors of this paper has a financial or personal relationship with other people or organizations that could inappropriately influence or bias the content of the paper.

## Authors’ contributions

MB conceived the study, participated in its design, sample collection, immunoassays and data analysis, and drafted the manuscript. KT participated in study design and sample collection, coordinated the farm site, and carried out immunoassays. TWM participated in study design and its coordination and helped to draft the manuscript. All authors read and approved the final manuscript.
